# Disentangling the Drivers of Diversity and Distribution of Fungal Community Composition in Wastewater Treatment Plants Across Spatial Scales

**DOI:** 10.3389/fmicb.2018.01291

**Published:** 2018-06-18

**Authors:** Haihan Zhang, Ji Feng, Shengnan Chen, Baoqin Li, Raju Sekar, Zhenfang Zhao, Jingyu Jia, Yue Wang, Pengliang Kang

**Affiliations:** ^1^Key Laboratory of Northwest Water Resources, Environment and Ecology, Ministry of Education, Xi’an University of Architecture and Technology, Xi’an, China; ^2^Shaanxi Key Laboratory of Environmental Engineering, Xi’an University of Architecture and Technology, Xi’an, China; ^3^Institute of Environmental Microbial Technology, Xi’an University of Architecture and Technology, Xi’an, China; ^4^Guangdong Key Laboratory of Integrated Agro-environmental Pollution Control and Management, Guangdong Institute of Eco-environmental Science and Technology, Guangzhou, China; ^5^Department of Biological Sciences, Xi’an Jiaotong-Liverpool University, Suzhou, China

**Keywords:** fungal community, activated sludge, ITS gene, network analysis, co-occurrence interactions

## Abstract

Activated sludge microbial community composition is a key bio-indicator of the sustainability of wastewater treatment systems. Therefore, a thorough understanding of the activated sludge microbial community dynamics is critical for environmental engineers to effectively manage the wastewater treatment plants (WWTPs). However, fungal communities associated with activated sludge have been poorly elucidated. Here, the activated sludge fungal community in 18 geographically distributed WWTPs was determined by using Illumina sequencing. The results showed that differences in activated sludge fungal community composition were observed among all WWTPs and also between oxidation ditch and anaerobic-anoxic-aerobic (A/A/O) systems. Ascomycota was the largest phyla, followed by Basidiomycota in all samples. Sporidiobolales and Pezizales were the most abundant order in oxidation ditch and A/A/O systems, respectively. The network analysis indicated cooperative and co-occurrence interactions between fungal taxa in order to accomplish the wastewater treatment process. *Hygrocybe* sp., *Sporobolomyces* sp., *Rhodotorula* sp., *Stemphylium* sp., *Parascedosporium* sp., and *Cylindrocarpon* sp., were found to have statistically significant interactions. Redundancy analysis revealed that temperature, total phosphorus, pH, and ammonia nitrogen were significantly affected the fungal community. This study sheds light on providing the ecological characteristics of activated sludge fungal communities and useful guidance for improving wastewater treatment performance efficiency.

## Introduction

In wastewater treatment systems, activated sludge microbial communities play vital roles in mediating the treatment efficiency by driving various biochemical processes and degrading diverse contaminants (Wagner et al., 2002). Microbial community composition associated with activated sludge is an important bio-indicator of the sustainability and health of wastewater treatment systems (Zhang et al., 2012). Therefore, a comprehensive assessment and understanding of the activated sludge microbial community dynamics is critical for environmental engineers to effectively manage the biological wastewater treatment facilities in wastewater treatment plants (WWTPs). Previous studies have extensively characterized the bacterial (Shchegolkova et al., 2016), archaeal (Gonzalez-Martinez et al., 2018), and protozoa (Siqueira-Castro et al., 2016) communities in activated sludge from full-scale WWTPs (Wang et al., 2012) and lab-scale bioreactors (Griffin and Wells, 2017). Nevertheless, fungal communities associated with activated sludge from different WWTPs have been poorly elucidated, and only few studies focused on the fungal communities in WWTPs (Evans and Seviour, 2012; Wei et al., 2018).

Fungal communities are critical components of activated sludge and act as the predominant decomposers in the wastewater treatment systems due to the vastness of fungal biomass and diversity, especially have a dominant role in organic matter biodegradation and nutrient cycling process (Evans and Seviour, 2012). Meanwhile, filamentous fungi have great relevance for sustainable activated sludge because several fungal species can cause sludge bulking and negatively affect wastewater treatment efficiency (Zheng et al., 2011). Thus, investigation of activated sludge fungal community composition in WWTPs may aid in sustainable wastewater treatment management (Zhang et al., 2012).

In the past few decades, the characteristics of activated sludge fungal communities were occasionally investigated by fluorescence *in situ* hybridization (Zheng et al., 2011), denaturing gradient gel electrophoresis, terminal restriction fragment length polymorphism profiles combined with cloning and sequencing (Evans et al., 2014), but these methods reveal only a fraction of the total fungal populations (Evans et al., 2014). Recently, Illumina Miseq sequencer producing unprecedented detail sequencing data was applied to shed light on a more exhaustive inventory and comparative analysis of fungal community structure (Niu et al., 2017). For instance, based on internal transcribed spacer (ITS) gene, Wei et al. (2018) recently determined the spatial variations of fungal communities in five WWTPs across three Chinese cities, and showed obvious spatial variations in fungal community distribution patterns. However, more comprehensive understanding of the shifts in fungal communities in the different WWTPs across a larger scale (e.g., different province) is quite limited, although geographic location is a primary driver for distribution of microbial diversity in different WWTPs (Wang et al., 2012; Zhang et al., 2012).

To date, despite high dimensionality microbial species sequence data are publicly available, assessing community datasets from ecological theory is still a big challenge to microbiologists. With the aid of advanced computational techniques, network analysis tools have been generated and widely used to elucidate the microbial community-scale network structure and taxonomic interaction in various ecosystems such as soils (Barberán et al., 2012; Xue et al., 2017), sediments (Li et al., 2016), aerosol particles (Bowers et al., 2012), extend the microbial community analyses beyond just the measurement and evaluation of diversity patterns. The resulting of co-occurrence association networks has been suggested to be more reliable and powerful to decipher the potential interactions between microbial taxa and their functional features in the systems (Barberán et al., 2012). Unfortunately, thus far, the co-occurrence patterns of fungal communities in activated sludge across large spatial gradients with WWTPs remain unclear.

To address this key knowledge gap, the present study was aimed at comprehensively characterizing the spatial distribution and co-occurrence patterns of activated sludge fungal communities from 18 full-scale WWTPs based on Illumina sequence platform and co-occurrence network analysis. The specific objectives were: (1) to investigate the diversity and structure of activated sludge fungal communities harbored in 18 WWTPs across different provinces in China; (2) to evaluate the co-occurrence patterns of these activated sludge fungal communities; (3) to find out the generalist fungal taxa (broadly distributed across WWTPs) and specialist (restricted to certain WWTPs); and finally (4) to explore the correlation between wastewater parameters and fungal community compositions. Overall, these results will provide valuable insight for understanding the characteristics of activated sludge fungal communities. Furthermore, improved insights from this study will help in advancing wastewater treatment system management based on the fungal community features.

## Materials and Methods

### Wastewater Treatment Plants and Sampling

In the present study, the tested WWTPs were located at different provinces of China: Shaanxi, Gansu, Guangdong, Fujian, Hubei, Shandong, Shanxi Province, and Tianjin City. Eighteen full-scale WWTPs were selected with total nitrogen removal efficiencies varied from 26 to 84%, in which the oxidation ditch and anaerobic-anoxic-aerobic (A/A/O) wastewater treatment processes were operated. Detailed characteristics of the 18 WWTPs are listed in Supplementary Figure 1 and **Table 1**. Briefly, BeiShiQiao (Abbrev. BSQ), HanSi (HS), LanZhou 1 (LZ1), LanZhou 2 (LZ2), WuWu (WW), and YangLing (YL) WWTPs were located in the west region of China, while GuangZhou (GZ), JinJiang (JJ), ShiShi (SS), WuHan (WH), XiaMen 1 (XM1), and XiaMen 2 (XM2) were located in the south area. LiaoCheng (LC), TianJin 1 (TJ1), and TianJin 2 (TJ2) were located in the east region and TaiYuan 1 (TY1), TaiYuan 2 (TY2), and TaiYuan 3 (TY3) were in the north. In each WWTPs, triplicate activated sludge samples were taken from the WWTP aeration tank in late November 2015 and the samples were transferred into 50 ml-sterile tubes (Kangning, China), put into a cooler which had a lower temperature of 8°C and then transported immediately to the laboratory. The fresh activated sludge samples were centrifuged for 10 min at 12,000 *g* (Eppendorf, Hamburg, Germany), and the labeled pellets were then stored at -20°C for further DNA extraction process. The wastewater physico-chemical properties of influent and effluent were determined from samples collected from each sampling sites.

**Table 1:** Fungal community diversity indices based on ITS gene of Illumina Miseq sequencing data from each activated sludge of the 18 wastewater treatment plants (WWTPs).

WWTPs^∗^	0.97 level					
	OTU	ACE	*Chao* 1	Coverage	Shannon (*H′*)	Simpson (*D*)
BSQ	72	86 (78,107)	83 (76,104)	0.999	0.2 (0.19,0.21)	0.95 (0.94,0.95)
GZ	225	232 (228,243)	231 (227,246)	0.999	2.51 (2.48,2.54)	0.20 (0.19,0.20)
HS	101	102 (101,107)	103 (101,116)	0.999	0.78 (0.76,0.8)	0.77 (0.77,0.80)
JJ	134	162 (148,189)	156 (144,186)	0.998	2.24 (2.22,2.26)	0.18 (0.18,0.19)
LC	24	44 (30,93)	50 (31,123)	0.999	0.1 (0.09,0.11)	0.97 (0.97,0.97)
LZ1	130	152 (140,175)	148 (137,177)	0.999	2.31 (2.3,2.33)	0.15 (0.15,0.16)
LZ2	41	282 (199,409)	118 (63,307)	0.999	1.42 (1.4,1.44)	0.44 (0.43,0.45)
SS	81	86 (83,98)	85 (82,99)	0.999	2.05 (2.03,2.06)	0.20 (0.19,0.20)
TJ1	124	128 (125,138)	128 (125,142)	0.999	2.32 (2.3,2.34)	0.21 (0.21,0.22)
TJ2	126	128 (126,134)	128 (126,138)	0.999	2.08 (2.06,2.1)	0.24 (0.24,0.25)
TY1	145	164 (155,185)	157 (150,176)	0.998	1.11 (1.1,1.13)	0.50 (0.50,0.51)
TY2	144	197 (174,240)	176 (159,211)	0.998	1.76 (1.74,1.78)	0.37 (0.36,0.37)
TY3	161	192 (177,220)	182 (170,208)	0.998	2.16 (2.14,2.19)	0.21 (0.20,0.20)
WH	103	111 (106,126)	109 (105,125)	0.999	1.9 (1.88,1.91)	0.30 (0.30,0.30)
WW	161	165 (162,174)	166 (162,180)	0.999	2.18 (2.15,2.2)	0.28 (0.28,0.29)
XM1	50	99 (76,142)	127 (72,316)	0.999	1.23 (1.21,1.24)	0.45 (0.45,0.46)
XM2	48	110 (81,164)	81 (59,144)	0.998	0.47 (0.46,0.49)	0.83 (0.83,0.84)
YL	94	104 (98,119)	101 (96,117)	0.999	1.44 (1.41,1.46)	0.44 (0.43,0.44)
West (*n* = 6)	100	148	120	0.999	1.39	0.51
East (*n* = 3)	91	100	102	0.999	1.50	0.47
North (*n* = 3)	150	184	172	0.998	1.68	0.36
South (*n* = 6)	107	133	132	0.999	1.73	0.36

*^∗^Abbrev. BSQ, BeiShiQiao; HS, HanSi; LZ1, LanZhou 1; LZ2, LanZhou 2; WW, WuWu; YL, YangLing; LC, LiaoCheng; TJ1, TianJin 1; TJ2, TianJin 2; TY1, TaiYuan 1; TY2, TaiYuan 2; TY3, TaiYuan 3; GZ, GuangZhou; JJ, JinJiang; SS, ShiShi; WH, WuHan; XM1, XiaMen 1; XM2, XiaMen 2.*

### Physico-Chemical Properties of Wastewater

The following physico-chemical properties of influent and effluent samples were determined: briefly, pH determination was performed by using a pH meter (Leici, Shanghai, China). Ammonia nitrogen (NH_4_^+^-N), biological oxygen demand (BOD_5_), total phosphorus (TP) were determined according to the standard methods (The State Environmental Protection Administration of China [SEPA], 2002). Suspended solids (SSs) were measured using the dry-weight of particles trapped by a filter (Larrarte and Pons, 2011).

### Extraction and Purification of Genomic DNA

The total microbial DNA was extracted from the AS samples to characterize the fungal communities (Gonzalez-Martinez et al., 2016), The genomic DNA was extracted from fresh activated sludge (1.0 g) using Soil DNA extraction kit (Omega Bio-tek, Norcross, GA, United States). The DNA extracts from the three replicates were pooled together for each WWTPs, and then purified using a DNA cleanup kit (Omega Bio-tek, Norcross, GA, United States). The purified DNA was checked by using a NanoDrop-2000 spectrophotometer at the absorption ratio of A260/A280 (Wang et al., 2012), and then the purified DNA samples were frozen (-20°C) for PCR (Gonzalez-Martinez et al., 2018).

### Illumina MiSeq Sequencing of Fungal ITS Gene

To determine the AS fungal community diversity and structure, the fungal ITS region of nuclear rDNA was sequenced using Miseq sequencer (Gonzalez-Martinez et al., 2018; Wei et al., 2018). Fungal ITS region was amplified using the fungal-specific primers ITS1-F (5′-CTTGGTCATTTAGAGGAAGTAA-3′) coupled with ITS2 (5′-GCTGCGTTCTTCATCGATGC-3′) described by Mueller et al. (2014), and the adapter sequences were added to the end of the 5′ primers (Wei et al., 2018; Zhang H. et al., 2018). PCR amplification was undertaken in mixtures (25 μL): 12.5 μL of 2× PCR Master mix (Tiangen, Beijing, China), 10 ng of template DNA, 10 μM of each primers, and ddH_2_O was added to balance the final volume. The thermal cycling protocol consisted of following amplification conditions: 5 min at 96°C, 28 cycles of 30 s at 97°C, 30 s at 61°C, 90 s at 72°C, and 10 min at 72°C for a final extension. PCR was carried out in triplicate on a C-1000 thermal cycler (Bio-Rad, United States). After successful amplifications, amplified-products were checked by 1.5% agarose gels electrophoresis and then purified using the PCR cleanup kits (Tiangen, Beijing, China) (Maza-Márquez et al., 2016; Zhang H. et al., 2018). The concentrations of purified PCR products were determined by a UV-Vis spectrophotometer (ND-2000, Thermo Fisher Scientific, Waltham, MA, United States). All sample libraries were combined in equimolar ratios (Sun et al., 2018) and the library quality was checked by the Bioanalyzer 2100. Subsequently, the paired-end sequencing of each amplicon library was sequenced following the manufacturer’s standard protocol (Sun et al., 2018; Zhang H. et al., 2018).

### Data Processing and Bioinformatics Analysis

To analyze the raw sequence data, quantitative insights into microbial ecology (QIIME) software package (version 1.7.0) was used to treat the fungal ITS sequences as described previously (Caporaso et al., 2010). The quality control and filtering of sequences were performed using MOTHUR pipeline (version 1.31.2) (Schloss et al., 2009). Briefly, after removing the primer and barcode sequences, chimeric sequences were discarded and short sequences less than 50 bp and sequence with any unknown nucleotides were also removed from the dataset (Wei et al., 2018). The number of raw reads was 455,526 after filtering and quality control. Subsequently, based on 97% identity, the reference dataset for fungal ITS sequences named UNITE-reference database was used to classify and taxonomically assign the high-quality reads into fungal operational taxonomic units (OTUs) (Kõljalg et al., 2013).

### Co-occurrence Network Analysis

The interactions between different fungal functional groups such as competition or cooperation, a co-occurrence network was characterized to reflect the co-occurrence of fungal community (Barberán et al., 2012; Lupatini et al., 2014). Relative abundances of less than 0.01% of the total OTUs were removed in order to reduce the rare OTUs. Average degree, the length of average path, numbers of nodes and edges, degree, network diameter, and clustering coefficient were also calculated (Barberán et al., 2012). Betweenness centrality was used to determine the most densely connected node in each module (Lupatini et al., 2014). Nodes with high degree (>100) was recorded as keystone fungal species in co-occurrence networks (Ma et al., 2016). A valid co-occurrence event should be Spearman’s correlation coefficient (SPSS version 20.0) (ρ > 0.6) combined with statistically significant at 0.01 level (Lupatini et al., 2014; Sun et al., 2018). Co-occurrence network was constructed and visualized by using the program GePhi (version 0.9.2)^1^ combined with the “ForceAtlas2” continuous graph layout algorithm (Barberán et al., 2012; Ma et al., 2016).

### Nucleotide Sequence Accession Number

The original sequences were deposited into the NCBI Sequence Read Archive (SRA) with Accession No. SRP079084.

### Statistical Analysis

One way-ANOVA was performed and followed by a Tukey’s HSD significant difference test using SPSS 20.0 software. For community diversity indices, *Chao*1, the abundance-base coverage estimator (ACE), Shannon index (*H′*), and Simpson index (*D*) were calculated by Mothur software package (version 1.31.2) (Schloss et al., 2009). The diversity coverage was also calculated using Good’s formula^2^. A heat map was generated using R (version 3.2.3) to show the relative abundance of dominant fungal community at genus level. Circos graphs for fungal community compositions were conducted using the online Circos software. Correlations between wastewater physico-chemical parameters and community diversity were generated using Spearman’s correlation. Redundancy analysis (RDA) combined with Monte Carlo test (999 permutations) was conducted to elucidate the linkage between fungal community compositions and physio-chemical variables of wastewater collected from different WWTPs. RDA was constructed in CANOCO for Windows (version 4.5).

## Results and Discussion

### Physico-Chemical Properties Wastewater

The wastewater physico-chemical properties of 18 WWTPs were summarized in Supplementary Tables 1, 2. WWTPs treated mainly domestic wastewater, except that GZ, LZ1, LZ2, TY1, TY2, TY3, and XM2 had proportion (10–30%) of industrial wastewater component. When the activated sludge samples were collected, the water temperature ranged from 10 to 25°C (Supplementary Table 2). Generally, the wastewater collected from all WWTPs was slightly alkaline. pH varied from 7.13 to 8.21 in the influent of WWTPs, and the system reduced the pH to values close to 6.93–8.14, which is consistent with previous report (Tanyol and Demir, 2016). However, pH ranged from 6.5 to 7.3 was also observed in other 18 WWTPs in China (Niu et al., 2017). Meanwhile, it was found that WWTPs reduced the concentrations of BOD_5_, ammonia nitrogen (NH_4_^+^-N) by 83 to 98 and 73 to 99%, respectively. The average TP removal efficiency was 87%, the WWTPs located in the west region had the significant higher TP removal efficiency (*P* < 0.05). The concentration of SSs varied from 56 to 684 mg/L in the influent of WWTPs, and the average removal efficiency of SS was 95%. Filamentous fungi have been demonstrated to remove the formation of heavier flocs, resulting in a lower turbidity of effluents (More et al., 2010). The average removal efficiency of NH_4_^+^-N in the present work was higher than that of previous research conducted by Wang et al. (2013), who observed a lower ammonia nitrogen removal efficiency (approximately 76%) in a municipal WWTP located at University of Cape Town, South Africa. Moreover, a previous study showed that the SS and BOD_5_ removal efficiencies were 87 and 93% respectively in a domestic WWTP in Turkey (Tanyol and Demir, 2016). In wastewater treatment ecosystems, fungal strains can remove phosphorus and nitrogen from wastewater (Hultberg and Bodin, 2017). Together, these results revealed that there were significant improvements in wastewater qualities after biochemical treatment process.

### Diversity of Activated Sludge Fungal Community

In total, 274,226,652 bp total bases of reads were obtained from the sequencer. 455,526 sequences with average length of 252 bp passed the quality control. As shown in **Table 1**, chimera filtering generated in 20,248 to 33,735 effective sequences per sample. Overall, a total of 1,964 OTUs were recorded and the OTUs ranged from 24 to 225 (**Table 1**). The diversity indices of fungal community had clear spatial separations. The highest ACE index was found in GZ with 232, and lowest of that was observed in LC with 44. Similarly, GZ also had the highest *Chao 1* index, which was 4.62 times higher than that of LC (**Table 1**). In contrast, LC had the highest Simpson index (*D*). Fungal diversity indices were highly variable across 18 WWTPs, however, no significant differences were observed in Good’s coverage (**Table 1**). The Good’s coverage (>99%) suggested that the depth of ITS gene sequence was enough to assess the fungal community diversity across all samples (Maza-Márquez et al., 2016). Similarly, low Shannon’s diversity index (*H′*) of fungi in a membrane bioreactor (*H′* = 1.56) was also reported by Maza-Márquez et al. (2016). Intriguingly, LC had the lowest OTUs number and diversity indices, meanwhile, the lowest TP removal efficiency was also found in LC with 66.67%. To a certain extent, this might be able to explain the phenomenon that TP removal efficiency of LC WWTP was lower than other WWTPs. However, no significant correlation was found between TP removal performance and fungal diversity (*P* > 0.05). The OTUs and ACE observed in this study were much lower than that of previous study (Niu et al., 2017) which found an average of 450 OTUs and 551 ACE per sample via 454 pyrosequencing. A possible explanation for this disparity is that Niu et al. (2017) used two fungal specific forward primers (ITS1-F and ITS2) combined with another reverse primer (A571R) to amplify ITS gene. Although 460,890 high quality sequences were obtained, which was slightly more than this work, much more OTUs were generated. Meanwhile, Wei et al. (2018) recently determined the fungal community in five WWTPs located in three different cities of China, and observed that 315,570 sequences of ITS gene was clustered into 2,469 OTUs. Changes in diversity patterns in fungal communities were observed between the samples collected from different WWTPs and cities. Shannon index (*H′*) ranged from 1.49 to 5.05, however, the present study exhibited a lower taxonomic diversity than previous evaluations (Niu et al., 2017; Wei et al., 2018). The most important reason is that primers are important for exploring the fungal community using high-throughput sequencing techniques. In this work, ITS1-F and ITS2 were used. Therefore, this study could be underestimating the diversity of the fungal populations due to the choice of the primers, with a lower number of OTUs obtained. More sequencing information of fungal community can be further investigated by targeting ITS and 18S rDNA regions.

### Structure and Composition of Activated Sludge Fungal Community

**FIGURE 1:**
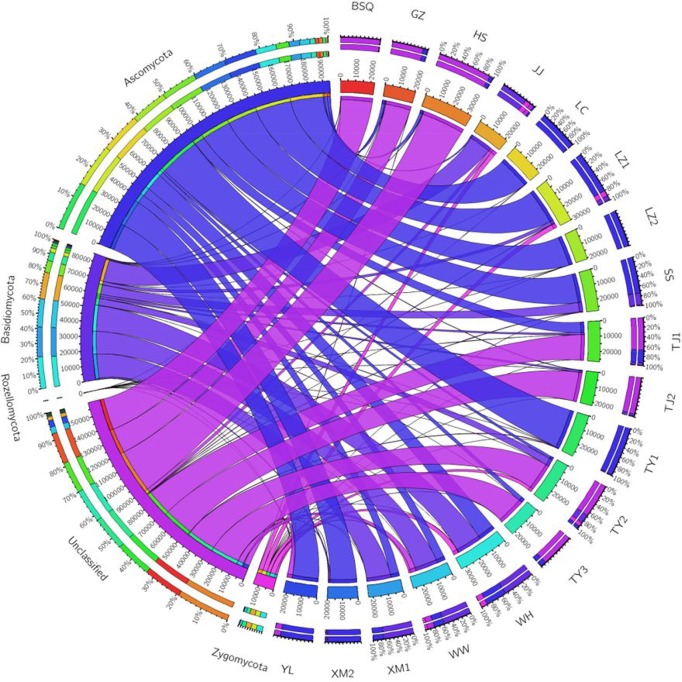
Circos representation of fungal communities at phylum level from the activated sludge of the 18 wastewater treatment plants (WWTPs).

Overall, a total of 4 phyla, 11 classes, 16 orders, 56 families, and 101 genera were identified in activated sludge samples collected from 18 WWTPs, while 150 OTUs were identified to the species level. Changes in fungal communities were analyzed at the phylum, class, order, and genus levels. **Figure 1** summarizes the relative abundance of phylum level fungal community in activated sludge from different WWTPs. Notably, Ascomycota was the most dominant phylum, accounting for approximately 43.44% of all phyla observed. Niu et al. (2017) reported the Ascomycota and Basidiomycota accounted for 51.82 and 42.94% of the fungal phyla observed in activated sludge collected from 18 WWTPs. In our study, LZ2 had the highest proportion of Ascomycota with 99.78%, followed by LC (99.10%), TY1 (98.21%), and XM2 (93.03%). Basidiomycota accounted for 18.3% of the fungal populations across all samples. The phylum Basidiomycota was found to be abundant in XM1 (73.50%), followed by WW (59.89%) and WH (53.67%). However, 35.44% proportion of OTUs was unclassified (**Figure 1**). Surprisingly, unclassified OTUs accounted for over 98% of the fungal reads in BSQ. The number of phyla and genera observed in the present study were lower than that of previous work by Niu et al. (2017), in which the phyla and genera observed were 7 and 195.

**FIGURE 2:**
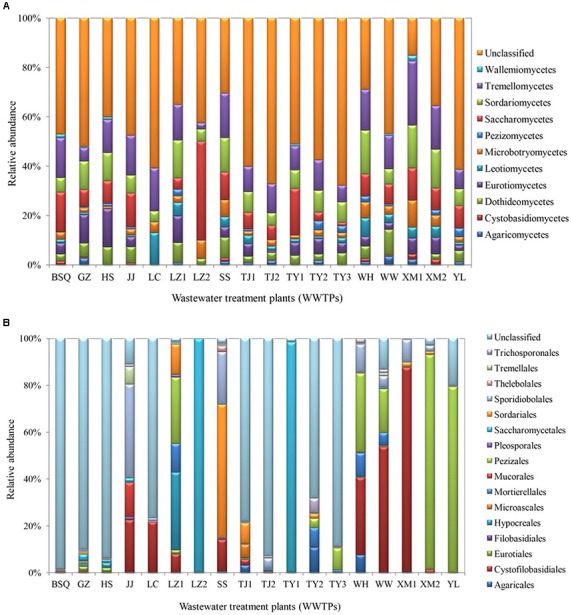
Fungal communities at **(A)** class and **(B)** order levels from the activated sludge of the 18 WWTPs.

The class-level taxonomic compositions of fungi in different samples analyzed in this study varied significantly. Tremellomycetes (24.01%) was the most dominant class, followed by Sordariomycetes (19.28%), Saccharomycetes (18.49%), and Eurotiomycetes (11.84%) (**Figure 2A**). The present result is consistent with the study by Wei et al. (2018), which showed that Saccharomycetes represented in high abundance in summer samples of Qingdao WWTP (93.73%) and winter samples of Qingdao WWTP (63.89%), while Tremellomycetes was dominant in other WWTPs including Qingdao WWTP (53.29%), Wuxi WWTP (34.20% in summer) and (29.25% in winter). A previous study demonstrated that cold environments favored Tremellomycetes and displayed higher diversity in the full-scale WWTPs sampled in the Polar Arctic climate zones (Gonzalez-Martinez et al., 2018). Saccharomycetes was also reported previously in a membrane bioreactor (Maza-Márquez et al., 2016).

**FIGURE 3:**
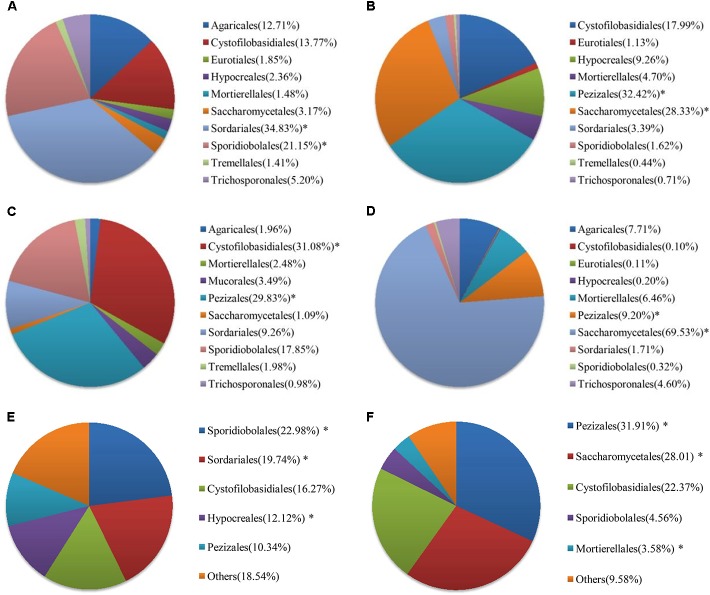
Fungal communities at order levels from the 18 WWTPs located at the **(A)** east (*n* = 3), **(B)** west (*n* = 6), **(C)** south (*n* = 6), and **(D)** north (*n* = 3) regions of China. Fungal communities at order levels from the 18 WWTPs with **(E)** oxidation ditch system (*n* = 4) and **(F)** A/A/O system (*n* = 14). ^∗^ Represents significant difference at *P* < 0.05 level.

At the order level, the proportion of fungal community composition differed strongly across the 18 WWTPs. Specifically, all WWTPs were dominated by Pezizales (15.66%), Saccharomycetales (12.53%), Cystofilobasidiales (12.33%), Sporidiobolales (5.44%), Sordariales (3.83%), Mortierellales (2.26%), Hypocreales (2.07%), and Agaricales (1.39%) (**Figure 2B**). TY1 and XM2 mixed domestic and industrial wastewater showed unique fungal communities. Saccharomycetales (97.97%) was dominant in TY1, while Pezizales (91.34%) in XM2 (**Figure 2B**). The microbial community can be shaped by wastewater composition (domestic wastewater mixed with different proportion of industrial wastewater), industrial wastewater usually contains several toxic elements such as heavy metals and various chemicals, which are harmful to the development of fungal species in wastewater treatment systems (Pramod et al., 2015). Interestingly, significant differences in fungal communities at order level were observed among the activated sludge samples collected from WWTPs in east, west, south, and north regions in China (**Figure 3**). In the east region, the dominant orders observed were Sordariales (34.83%), Sporidiobolales (21.15%), and Cystofilobasidiales (13.77%) (**Figure 3A**). In the west region, the most dominated fungal community was Pezizales (32.42%), followed by Saccharomycetales (28.33%), and Cystofilobasidiales (17.99%) (**Figure 3B**). However, Saccharomycetales (69.53%) was predominant in the north region (**Figure 3D**) and Cystofilobasidiales (31.08%) and Pezizales (29.83%) were dominant in the south region (**Figure 3C**). The relative abundances of Sporidiobolales (22.98%), Sordariales (19.74%), and Hypocreales (12.12%) (*P* < 0.05 in all case) in oxidation ditch were significantly higher than that of A/A/O systems (**Figure 3E**). In contrast, in the A/A/O systems, Pezizales (31.91%), Saccharomycetales (28.01%), Mortierellales (3.58%) (*P* < 0.05 in all case) were the dominant orders (**Figure 3F**). This is consistent with previous evidences that wastewater treatment types (A/A/O, oxidation ditch, and membrane bioreactor) can substantially restructure activated sludge microbial community composition through dramatic differences in treatment operational properties (Hu et al., 2012). Moreover, the previous study demonstrated that microbial community composition in oxidation ditches was more divers than in A/A/O systems (Hu et al., 2012), therefore, the structure of microbial community can be structured by different wastewater treatment processes (Baek and Pagilla, 2009). Moreover, Evans et al. (2014) suggested that the observed differences in the fungal community structures was due to the different wastewater characteristics or operational parameters in the WWTPs. It is therefore that the specific operational parameters [e.g., retention time, plant dimension, temperature, food to microorganisms (F/M) ratio] can be regulated to achieve the desired treatment performance aims (Niu et al., 2015).

**FIGURE 4:**
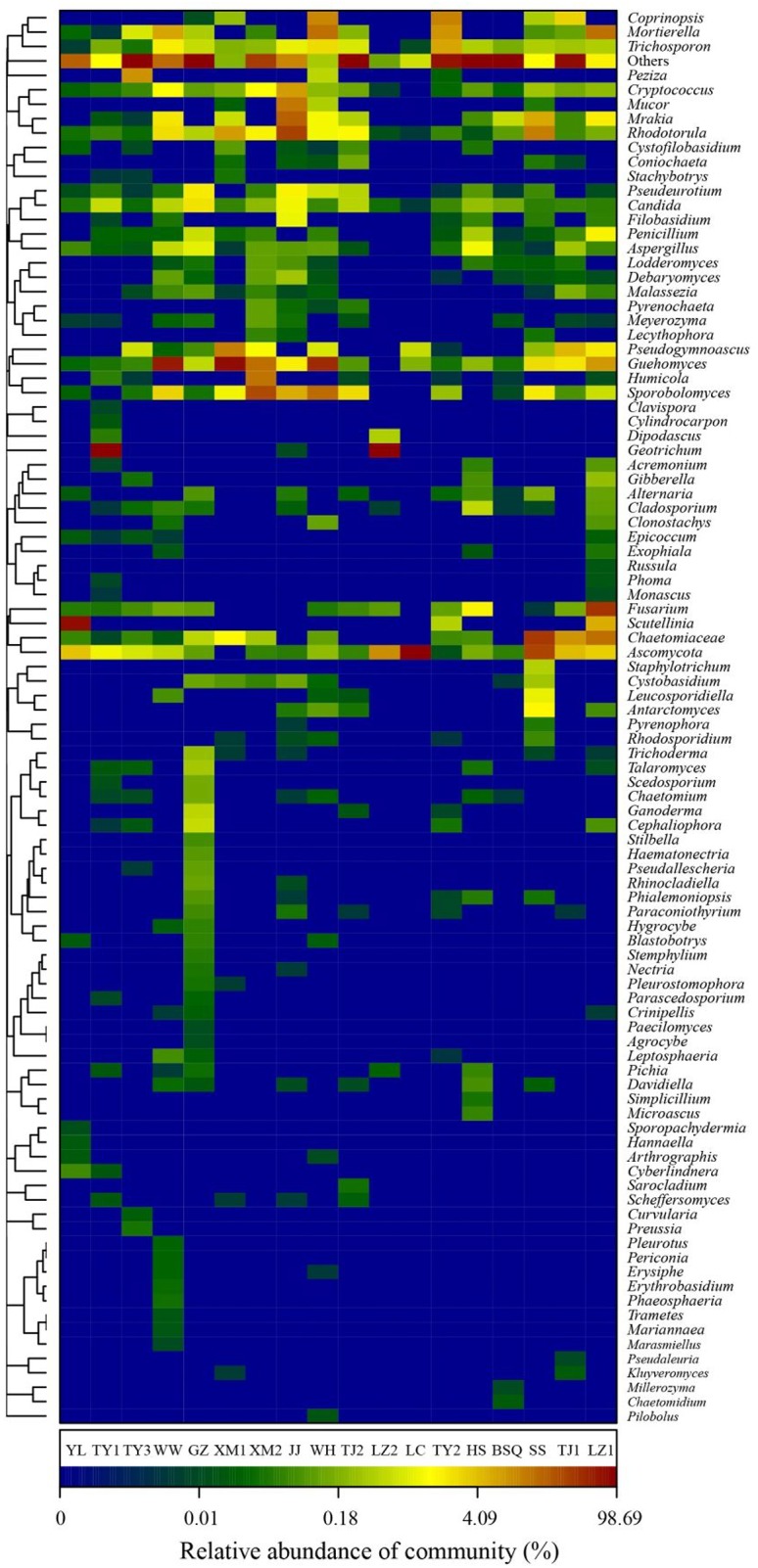
Heat map fingerprinting of fungal communities at genus level (top 100 genera) based on ITS gene of Illumina Miseq sequencing data from each activated sludge of the 18 WWTPs.

Further classification at the genus level, a hierarchical heat map of the relative abundance of top 100 abundant fungal genera is shown in **Figure 4**. Generally, heat map fingerprints indicated that fungal communities in each WWTPs were unique. For instance, the most dominant genera in the plants like WW, XM1, and WH was *Guehomyces*, accounting for 63, 76, and 48% of the total OTUs observed, respectively. High level of *Ascomycota* (98%) was observed in LC. *Chaetomiaceae* (relative abundance of total OTUs, 37%) in SS. *Scutellini*a (76%) in YL. *Geotrichum* (96%) in TY1. and 90% in LZ2. Notably, *Candida* and *Guehomyces* were the abundant genera in all samples. Thirteen fungal genera, accounting for 55.1% of the total sequences, were shared by WWTPs. Evans and Seviour (2012) used PCR-DGGE to examine the biodiversity of fungi in AS communities based on 18S rRNA genes, and the results showed that *Mucor* sp., *Cladosporium* sp., *Aspergillus* sp., and *Penicillium* sp., were the commonly observed fungal genera. *Candida* sp. (approximately 2.42%) was reported to be widely distributed in WWTPs (Wei et al., 2018). The *Candida* sp. was reported to involve in the removal of chromium and degrade phenol (Adav et al., 2007; Ye et al., 2010). Some species of *Geotrichum* sp. showed decolorization ability (Ayed et al., 2016). Previous reports indicated the occurrence of *Acremonium* sp.*, Rhodotorula* sp.*, Candida* sp., *Geotrichum* sp.*, Cladosporium* sp.*, Sporothrix* sp., and *Trichophyton* sp. in wastewater and activated sludge from WWTPs using culture-dependent method (Kacprzak et al., 2005), and the study showed that several genera (e.g., *Acremonium* sp., *Geotrichum* sp., and *Mucor* sp.) observed were human opportunistic pathogenic fungi (Maza-Márquez et al., 2016). *Geotrichum* sp., *Mucor* sp., and *Penicillium* sp., have been recorded previously as cycloheximide-resistant fungi in activated sludge (Awad and Kraume, 2011), therefore, these fungi may be harmful to wastewater treatment workers. As wastewater acts as medium for fungal growth, *Penicillium* accounts for 52% of treated wastewater, *Penicillium sclerotium*, *P*. *spinulosum*, and *P*. *granulatum* occurred most commonly in WWTPs and constructed wetlands (Kacprzak et al., 2003). *Aspergillus* sp. and *Trichoderma* sp. were the commonly occurring genus in WWTPs, *Penicillium corylophilum* and *Aspergillus niger* were accounting 98% of existing fungi in the wastewater treatment systems (More et al., 2010). However, in our study, *Microthrix* sp. was not detected. Because *Microthrix parvicella* was responsible for bulking (Rossetti et al., 2005). Thus, these results confirmed that the activated sludge ecosystems are stable.

A previous study showed that oleaginous yeast was used in wastewater treatment process and the fungal genus *Trichosporon* sp. was found to degrade suspended-solids and remove caffeine (Lakshmi and Das, 2013). It was also reported that *Trichosporon asahii* was the dominant filamentous fungi in the AS, and caused activated sludge fungal bulking (Zheng et al., 2011). *Rhodotorula* sp., is good at degrading toxic phenolic compounds in olive mill wastewater (Jarboui et al., 2012). Additionally, a cyanide resistant fungus, *Fusarium* sp., was used for the bioremediation of wastewater containing free cyanide in gold mine wastewater (Akinpelu et al., 2017). Moreover, *Fusarium* sp., was isolated from WWTP in Germany, and had cycloheximide-resistant ability (Awad and Kraume, 2011). *Pseudogymnoascus* sp., and *Aspergillus* sp. can effectively remove phenolic and polycyclic aromatic hydrocarbon compounds from highly alkaline industrial wastewater because the strains favor both laccase and manganese peroxidase activities (Batista-García et al., 2017). Moreover, potential use of filamentous fungal species for sludge treatment have also been critically analyzed and reviewed by More et al. (2010) and their study demonstrated that numerous filamentous fungi can degrade toxic compounds with bio-flocculation capacity.

### Core Fungal Communities

While determining the WWTPs generalists and specialists fungal community across 18 samples, we found that the core fungal communities, and populations in different WWTPs were highly dynamic. Among these, Ascomycota was the highly dominant and core phylum observed in LC, LZ2, and YL. At family level, Cystofilobasidiaceae was the core fungal taxa observed in BSQ with 74.4%, WW with 87.4%, and XM1 with 84.1%. The specialist fungal taxa in YL and LC were Pyronemataceae (99.4%) and Myxotrichaceae (75.0%) (**Table 2**). The group of specialists for Pezizaceae comprised 80.3% in TY3, while specialists for TJ2 were Trichosporonaceae (36.29%). Previously, it was demonstrated that core OTUs in influent wastewater from Polar Arctic Circle full-scale WWTPs with process temperature ranged from 3 to 6°C was belonged to Trichosporonaceae family (Gonzalez-Martinez et al., 2018). Collectively, these data further confirmed that different WWTPs clearly lead to the development of a locally abundant and specialized fungal community. In addition, these specialized fungal communities might be developed as wastewater treatment system health indicators because they are distributed extensively in activated sludge (Wei et al., 2018). These results are in agreement with a recent study by Fang et al. (2018) indicating that the geographically distributed WWTPs might share a core microbial community observed by Illumina Miseq sequencing data.

**Table 2:** Specialist fungal taxa (restricted to certain WWTPs) based on top 22 dominant fungal taxa from each activated sludge of the 18 wastewater treatment plants (WWTPs).

WWTPs^∗^	Relative abundance of specialist fungal taxa at family level
BSQ	Cystofilobasidiaceae (74.4%)
GZ	Pseudeurotiaceae (26.7%)
HS	Nectriaceae (36.5%)
JJ	Mucoraceae (34.9%)
LC	Myxotrichaceae (75.0%)
LZ1	Nectriaceae (43.9%)
LZ2	Dipodascaceae (99.9%)
SS	Chaetomiaceae (73.5%)
TJ1	Chaetomiaceae (42.6%)
TJ2	Trichosporonaceae (36.3%)
TY1	Dipodascaceae (99.7%)
TY2	Psathyrellaceae (38.7%)
TY3	Pezizaceae (80.3%)
WH	Cystofilobasidiaceae (60.2%)
WW	Cystofilobasidiaceae (87.4%)
XM1	Cystofilobasidiaceae (84.1%)
XM2	Cystofilobasidiaceae (42.4%)
YL	Pyronemataceae (99.4%)

*^∗^Abbrev. BSQ, BeiShiQiao; GZ, GuangZhou; HS, HanSi; JJ, JinJiang; LC, LiaoCheng; LZ1, LanZhou 1; LZ2, LanZhou 2; SS, ShiShi; TJ1, TianJin 1; TJ2, TianJin 2; TY1, TaiYuan 1; TY2, TaiYuan 2; TY3, TaiYuan 3; WH, WuHan; WW, WuWu; XM1, XiaMen 1; XM2, XiaMen 2; YL, YangLing.*

### Network Description of Fungal Taxa

**FIGURE 5:**
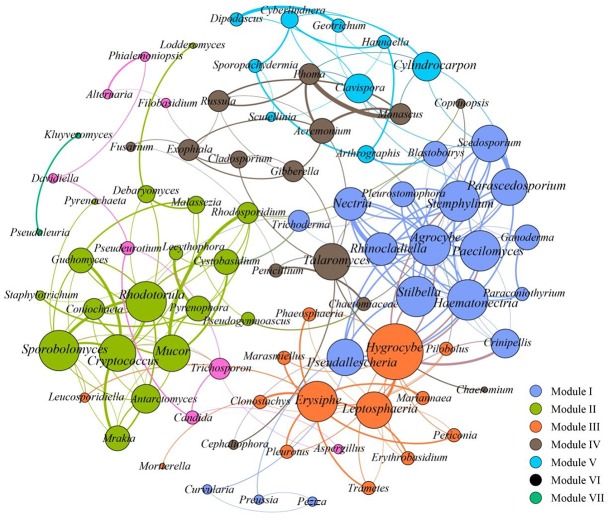
Co-occurrence network of the dominant fungal communities at genus level based on ITS gene of Illumina Miseq sequencing data from each activated sludge of the 18 WWTPs. A connection stands for a strong (Spearman’s ρ > 0.6) and significant (*P* < 0.01) correlations. Seven modules were generated, and the same color means the potential co-occurrence of fungal genera in the same module. The size of nodes represents the relative abundance of fungal OTUs.

**FIGURE 6:**
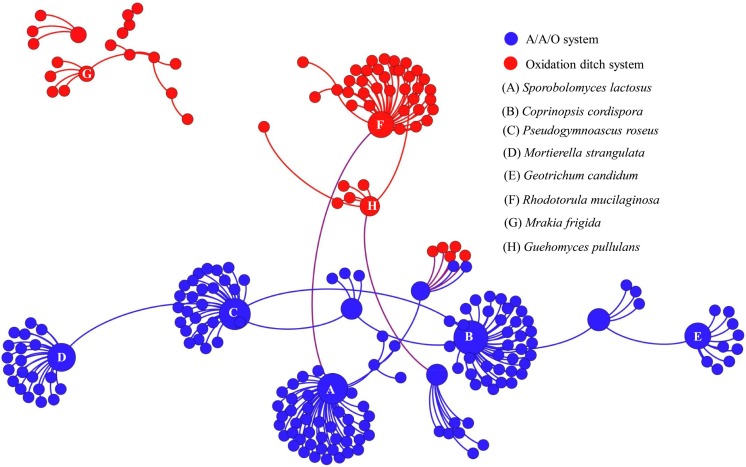
Co-occurrence network of the dominant fungal communities at species level based on ITS gene of Illumina Miseq sequencing data from each activated sludge of the 18 WWTPs. The same color means the potential co-occurrence of fungal species in the same system. Each node represents the fungal OTUs.

To further assess the potential interactions among fungal community members, network analysis was conducted. In total, 97 dominant fungal genera were used for co-occurrence network analysis after removal of the unclassified genera and several other genera which showed low relative abundance. Co-occurring fungal species might be essential to maintain the critical stability and activity of activated sludge. The network is compartmentalized into seven discrete modules of closely associated fungi, and each module is indicated by color. Strong and significant correlations (*P* < 0.01) are showed in **Figure 5**. The generated networks had 85 nodes (i.e., fungal genera). The general structures of co-occurrence networks included an average path length of 5.907, an average clustering coefficient of 0.134, a modularity index of 0.644, and an network diameter of 16. Nodes represent fungal genera, whereas edges represent significant positive correlations between pairs of genera. Modules I, II, and III had 19, 15, and 14 nodes. The larger size of nodes represent the more important genera in the fungal community (Sun et al., 2018). A highly connected fungal taxa in each module was defined as a “hub,” connectors and network hubs can be considered as keystone taxa due to their central position in a microbial network. Network modules and hubs in fungal community were shown in **Figure 5**. Interestingly, members of *Hygrocybe* sp., *Sporobolomyces* sp., *Rhodotorula* sp., *Stemphylium* sp., *Parascedosporium* sp., and *Cylindrocarpon* sp., were found to have statistically significant interactions (**Figure 5**) and the results suggests that these taxa are primary organisms involved in the removal of pollutants. At the same time, a clear separation in the co-occurrence analysis between A/A/O and oxidation ditches systems were shown in **Figure 6**. The direct interactions revealed that *Sporobolomyces lactosus*, *Coprinopsis cordispora*, *Mortierella strangulata* coexisted with 99 species in A/A/O system, while *Rhodotorula mucilaginosa*, *Mrakia frigida* were correlated with 49 species in oxidation ditches system. The results further revealed that the correlations of fungal species-species could be more critical than those dominant one in removing contaminant in wastewater treatment process. For instance, several dominate fungal populations drive the nutrients (e.g., BOD_5_ and NH_4_^+^-N) removal in wastewater treatment systems (Supplementary Figure 2). In general, the results suggest that the cooperative and co-occurrence interactions between different fungal species improve our understanding of activated sludge-fungal partnership in the wastewater treatment systems. Therefore, one of the research directions is to focus on exploring the physiology of these “hub” fungal species and the interactions within the fungal network could be crucial for both sustaining activated sludge health and ecosystem functioning which could provide a potential objective for novel wastewater treatment management strategies in the future.

### Linking Activated Sludge Fungal Communities to Wastewater Properties and Geographic Locations

**FIGURE 7:**
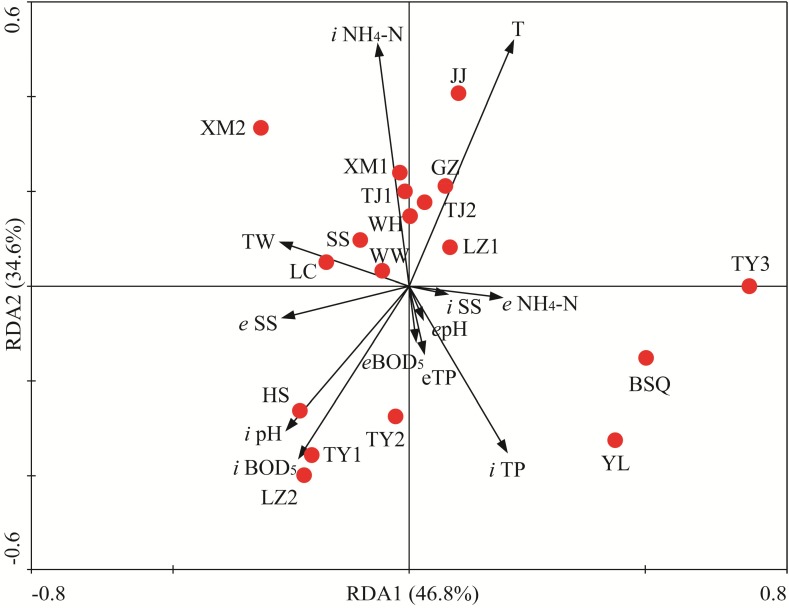
Redundancy analysis (RDA) of the linkage between fungal community compositions and wastewater physiochemical variables of 18 WWTPs. BOD_5_, biological oxygen demand; TP, total phosphorus; SSs, suspended solids; T, temperature; TW, type of wastewater. The first two RDA (RDA1 and RDA2) explained 46.8 and 34.6% of total variances, respectively. “*i*” and “*e”* represent “influent” and “effluent,” respectively.

To identify the most important factors likely influencing the fungal community composition, RDA was applied to determine the linkages between statistically significant wastewater parameters and fungal populations. The multivariate analysis of variance suggested that the fungal communities observed in 18 WWTPs were statistically different. RDA1 and RDA2 explained 46.8 and 34.6% of total variances, respectively (**Figure 7**). RDA biplot indicated that spatial and wastewater parameters variations significantly altered fungal communities. Monte Carlo permutation test also revealed that temperature, BOD_5_ (inflow), ammonia nitrogen and TP (inflow), pH significantly affected the fungal community (**Figure 7**, *P* < 0.05 in all cases). Based on the RDA results, BSQ and YL were located in the fourth quadrant whereas HS, LZ2, TY1, and TY2 were located in the third quadrant, which mainly structured by pH and BOD_5_. Temperature had significant influence on fungal communities associated with JJ, GZ, TJ2, and LZ1 (**Figure 7**). Temperature, an important physical parameter, can significantly affect the microbial community in WWTPs, the wastewater treatment efficiency (Gnida et al., 2016). In this study, temperature had significant influence on structure of fungal community (**Figure 7**). For instance, GZ had the highest OTUs with the highest temperature. The abundance of Sporidiobolales had significant positive correlation with temperature (*P* < 0.05). According to previous study, lower temperature (e.g., along with the decrease of temperature from summer to winter) has been demonstrated to reduce the diversity and shift the structure of microbial community (Flowers et al., 2013). Zhang B. et al. (2018) recently reported that activated sludge microbial community became more complicated with temperature increasing in four WWTPs used A/A/O and oxidation ditch processes. The limitation of present work is that the fungal community was investigated in November. Therefore, the seasonal variation of fungal community composition should be further explored in the future. The present results is also consistent with a previous research in which significant spatial variations were observed in samples collected from five WWTPs in China (Wei et al., 2018) and the results suggest correlation of the geographical distance and TP with activated sludge fungal community. The finding from Maza-Márquez et al. (2016) suggested that pH was the primary determinant of fungal population in membrane bioreactors. However, Xu et al. (2018) recently demonstrated that pH is not the most crucial factor regulating the community composition.

Activated sludge harbors highly diverse fungi that might be assembled differently among types of wastewater, chemical compositions, and operational parameters (Evans et al., 2014). The unique fungal community in different WWTPs could be due to several possible drivers, geographical location, temperature, season, nutrient load, type of wastewater, and percentage of industrial wastewater. The distinct spatial separation might be demonstrated that the local environmental factors (altitude, ultraviolet radiation) had influence in structuring the fungal communities (Niu et al., 2017; Fang et al., 2018). Similar results have also been showed that latitude and water temperature were regarded as the important factors that affect microbial community diversity in oxidation ditch system for domestic wastewater treatment (Xu et al., 2017). Similarly, a survey in 18 full-scale municipal WWTPs observed that the C/N ratio and dissolved oxygen (DO) were also found to be the most dominant drivers to the shifts of fungal community composition (Niu et al., 2017). These findings support the present study that spatial combined with wastewater parameters drives the extremely diverse fungal communities associated with the activated sludge.

## Conclusions

Differences in fungal community structure was observed among all WWTPs and between oxidation ditch and A/A/O systems. Ascomycota was the most dominant phylogenetic group, followed by Basidiomycota. *Hygrocybe* sp., *Sporobolomyces* sp., *Rhodotorula* sp., *Stemphylium* sp., and *Parascedosporium* sp. have statistically significant interactions in co-occurrence network, while fungal interactions may contribute to activated sludge functions more than species diversity. RDA indicated that spatial and wastewater parameters variations significantly structured fungal communities. These results provide valuable insight for understanding the characteristics of activated sludge fungal communities.

## Author Contributions

HZ and SC contributed to the experiment design. JF and ZZ performed the DNA extraction and amplification. YW, JJ, PK, and BL performed the statistical analysis. RS revised and polished the language. All authors contributed to manuscript revision and read and approved the submitted version.

## Conflict of Interest Statement

The authors declare that the research was conducted in the absence of any commercial or financial relationships that could be construed as a potential conflict of interest.

## Footnotes

1 https://gephi.github.io/

2 http://www.mothur.org/wiki/Coverage
