# Supplementary material for: Disentangling the Drivers of Diversity and Distribution of Fungal Community Composition in Wastewater Treatment Plants Across Spatial Scales
Source: Front Microbiol. 2018 Jun 18;9:1291. doi: 10.3389/fmicb.2018.01291 (PMC6015911; doi:10.3389/fmicb.2018.01291)
Supplement: Supplementary file 1 [file Data_Sheet_1.doc]

**Supplementary Materials**

**Disentangling the drivers of diversity and distribution of fungal community composition in wastewater treatment plants across spatial scales**

Haihan Zhang ^1,2,3*^, Ji Feng ^1,2,3^, Shengnan Chen ^1,2,3^, Baoqin Li ^4^, Raju Sekar ^5^, Zhenfang Zhao^1,2,3^, Jingyu Jia ^1,2,3^, Yue Wang ^1,2,3^ and Pengliang Kang ^1,2,3^

*^1^Key Laboratory of Northwest Resource, Environment and Ecology, MOE, Xi’an University of Architecture and Technology, Xi’an 710055, Shaanxi Province, China*

*^2^Shaanxi Key Laboratory of Environmental Engineering, Xi’an University of Architecture and Technology, Xi’an 710055, Shaanxi Province, China*

*^3^ Institute of Environmental Microbial Technology, Xi’an University of Architecture and Technology, Xi’an 710055, Shaanxi Province, China*

*^4^Guangdong Key Laboratory of Integrated Agro-environmental Pollution Control and Management, Guangdong Institute of Eco-Environmental Science and Technology, Guangzhou 510650, Guangdong Province, China*

*^5^Department of Biological Sciences, Xi'an Jiaotong-Liverpool University, Ren'ai Road 111, Suzhou 215123, Jiangsu Province, China*

*Corresponding author

Tel. +86-29-82202854;

Fax: +86-29-82202729;

Email: zhanghaihan@xauat.edu.cn

**TABLE 1.** The location and operational parameter characteristics of wastewater quality in eighteen wastewater treatment plants (WWTPs).

| **WWTPs*** | **Location** | **BOD_5_ (mg/L)** | | **NH_4_-N (mg/L)** | | **TP (mg/L)** | | **pH** | | **SS (mg/L)** | | **Treatment process** |
| --- | --- | --- | --- | --- | --- | --- | --- | --- | --- | --- | --- | --- |
|  |  | Influent | Effluent | Influent | Effluent | Influent | Effluent | Influent | Effluent | Influent | Effluent |  |
| BSQ | Shaanxi | 93 | 7 | 23 | 2 | 9.32 | 0.4 | 7.4 | 7.1 | 164 | 7 | Oxidization ditch |
| GZ | Guangdong | 120 | 20 | 30 | 8 | 3.5 | 1.5 | 7.13 | 7.10 | 150 | 20 | A/A/O |
| HS | Shaanxi | 186 | 13 | 28 | 3.4 | 5.45 | 0.2 | 7.5 | 7.2 | 138 | 9 | A/A/O |
| JJ | Fujian | 31.1 | 3.5 | 11.8 | 0.2 | 1.29 | 0.35 | 7.28 | 6.93 | 56 | 9 | Oxidization ditch |
| LC | Shandong | 160 | 8 | 40 | 0.8 | 3.0 | 1.0 | 8.12 | 7.72 | 200 | 15 | A/A/O |
| LZ1 | Gansu | 496 | 15.5 | 45.7 | 14.7 | 9.75 | 0.52 | 7.45 | 7.27 | 684 | 16 | Oxidization ditch |
| LZ2 | Gansu | 54 | 4 | 31 | 1.3 | 6.5 | 0.96 | 7.71 | 7.67 | 175 | 11 | A/A/O |
| SS | Fujian | 55 | 2.8 | 13.42 | 0.37 | 1.7 | 0.12 | 7.2 | 7.1 | 122 | 8 | Oxidization ditch |
| TJ1 | Tianjin | 75 | 4 | 38.9 | 1.6 | 5.12 | 0.3 | 7.87 | 7.34 | 154 | 3 | A/A/O |
| TJ2 | Tianjin | 64 | 7 | 37.4 | 1.2 | 4.27 | 0.46 | 7.93 | 7.16 | 169 | 8 | A/A/O |
| TY1 | Shanxi | 186 | 6.8 | 45.5 | 1.6 | 5.23 | 0.1 | 7.53 | 7.45 | 145 | 5.4 | A/A/O |
| TY2 | Shanxi | 184 | 4.7 | 32.4 | 1.2 | 2.84 | 0.68 | 8.21 | 8.14 | 220 | 4.8 | A/A/O |
| TY3 | Shanxi | 170 | 6.0 | 38.3 | 0.9 | 5.36 | 0.93 | 7.60 | 7.41 | 140 | 5.2 | A/A/O |
| WH | Hubei | 52 | 0.9 | 10.5 | 0.3 | 3.03 | 0.3 | 8.20 | 7.95 | 68 | 2 | A/A/O |
| WW | Shaanxi | 156 | 6.9 | 54 | 3.6 | 8.31 | 0.3 | 7.3 | 7.0 | 139 | 6.6 | A/A/O |
| XM1 | Fujian | 63 | 6 | 29 | 0.11 | 5.93 | 0.35 | 7.52 | 7.41 | 248 | 5 | A/A/O |
| XM2 | Fujian | 132 | 2 | 29.1 | 1.65 | 3.87 | 0.65 | 7.43 | 7.41 | 106 | 5 | A/A/O |
| YL | Shaanxi | 94 | 5.3 | 22.5 | 3.3 | 6.89 | 0.53 | 7.31 | 7.21 | 170 | 3 | A/A/O |

*Abbreviations: BeiShiQiao (Abbrev. BSQ), HanSi (HS), LanZhou 1 (LZ1), LanZhou 2 (LZ2), WuWu (WW), YangLing (YL), LiaoCheng (LC), TianJin 1 (TJ1), TianJin 2 (TJ2), TaiYuan 1(TY1), TaiYuan 2 (TY2), TaiYuan 3 (TY3), GuangZhou (GZ), JinJiang (JJ), ShiShi (SS), WuHan (WH), XiaMen 1 (XM1), XiaMen 2 (XM2). Biological oxygen demand: BOD_5_. Total phosphorus: TP. Suspended solids: SS. Anaerobic-anoxic-aerobic: A/A/O.

**TABLE 2.** The type of wastewater and temperature in eighteen wastewater treatment plants (WWTPs).

| **WWTPs*** | **Type of wastewater** | **Average temperature (℃)** |
| --- | --- | --- |
|
| BSQ | Domestic wastewater | 12 |
| GZ | 80% Domestic wastewater; 20% Industrial wastewater | 25 |
| HS | Industrial wastewater | 15 |
| JJ | Domestic wastewater | 22 |
| LC | Domestic wastewater | 14 |
| LZ1 | 90% Domestic wastewater; 10% Industrial wastewater | 10 |
| LZ2 | 90% Domestic wastewater; 10% Industrial wastewater | 11 |
| SS | Domestic wastewater | 23 |
| TJ1 | Domestic wastewater | 13 |
| TJ2 | Domestic wastewater | 12 |
| TY1 | 70% Domestic wastewater; 30% Industrial wastewater | 15 |
| TY2 | 70% Domestic wastewater; 30% Industrial wastewater | 16 |
| TY3 | 70% Domestic wastewater; 30% Industrial wastewater | 15 |
| WH | Domestic wastewater | 14 |
| WW | Domestic wastewater | 12 |
| XM1 | Domestic wastewater | 24 |
| XM2 | 80% Domestic wastewater; 20% Industrial wastewater | 25 |
| YL | Domestic wastewater | 13 |

*Abbreviations: BeiShiQiao (Abbrev. BSQ), HanSi (HS), LanZhou 1 (LZ1), LanZhou 2 (LZ2), WuWu (WW), YangLing (YL), LiaoCheng (LC), TianJin 1 (TJ1), TianJin 2 (TJ2), TaiYuan 1(TY1), TaiYuan 2 (TY2), TaiYuan 3 (TY3), GuangZhou (GZ), JinJiang (JJ), ShiShi (SS), WuHan (WH), XiaMen 1 (XM1), XiaMen 2 (XM2). Biological oxygen demand: BOD_5_. Total phosphorus: TP. Suspended solids: SS. Anaerobic-anoxic-aerobic: A/A/O.


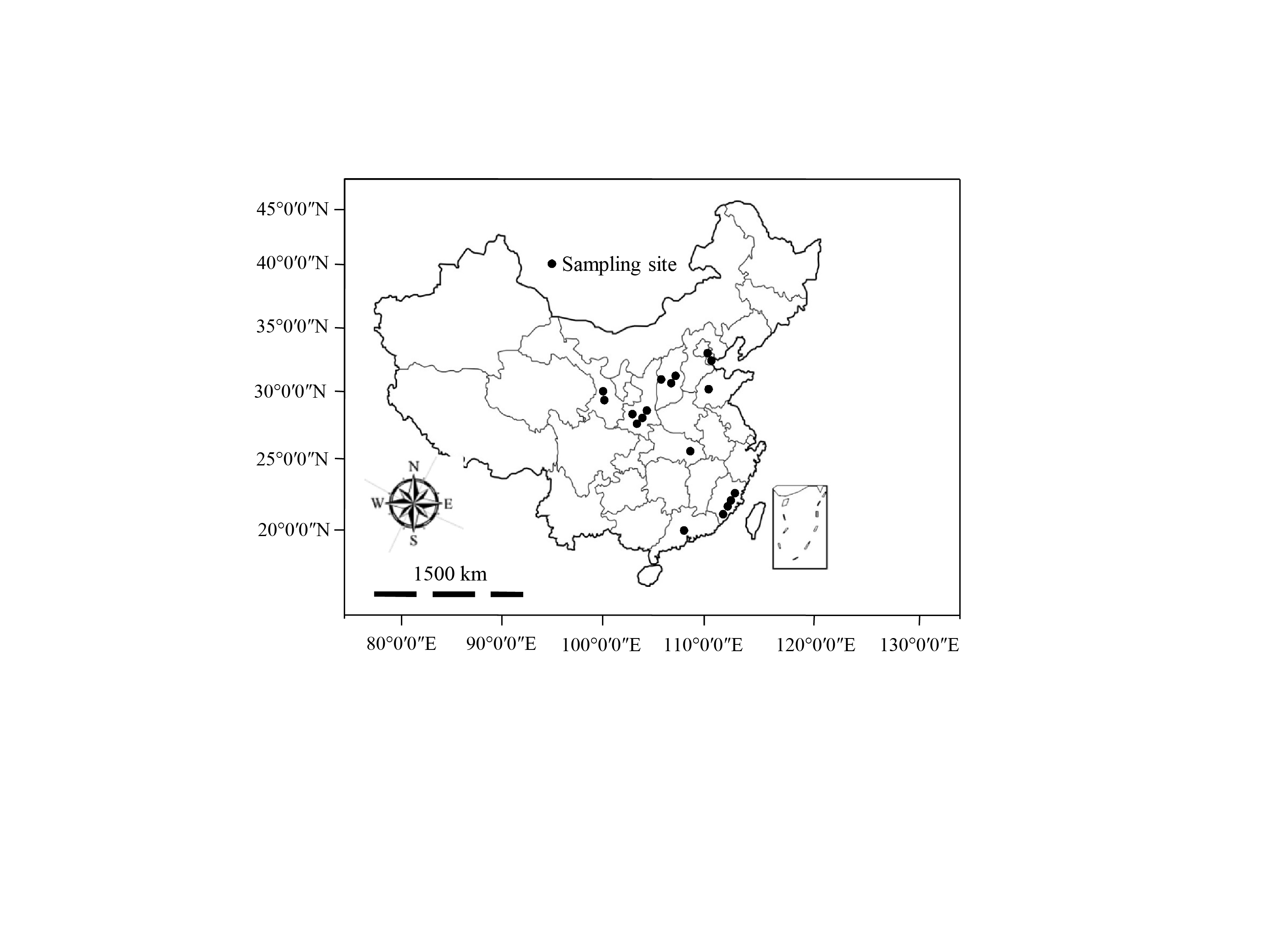


**FIGURE 1.** The sampling site map of the 18 geographically distributed WWTPs including BSQ, GZ, HS, JJ, LC, LZ1, LZ2, SS, TJ1, TJ2, TY1, TY2, TY3, WH, WW, XM1, XM2 and YL.


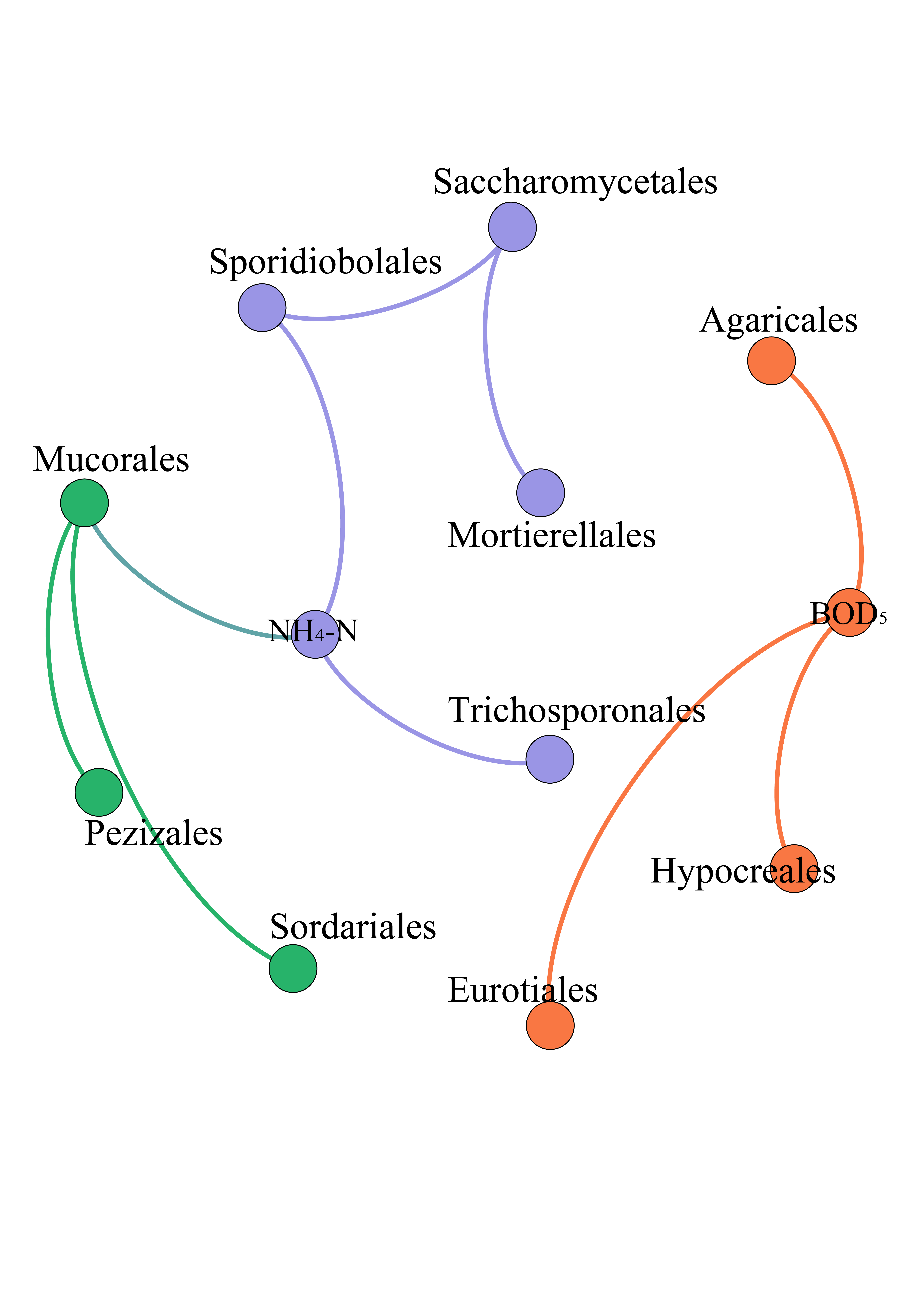


**FIGURE 2.** Network of the dominant fungal communities at order level. A connection stands for a strong (Spearman's ρ>0.6) and significant (*P* <0.01) correlations between BOD_5_, NH_4_^+^-N and fungal community.
